# Propolis Induces Chondroitin/Dermatan Sulphate and Hyaluronic Acid Accumulation in the Skin of Burned Wound

**DOI:** 10.1155/2013/290675

**Published:** 2013-03-07

**Authors:** Pawel Olczyk, Katarzyna Komosinska-Vassev, Katarzyna Winsz-Szczotka, Jerzy Stojko, Katarzyna Klimek, Ewa M. Kozma

**Affiliations:** ^1^Department of Community Pharmacy, Medical University of Silesia, ul. Kasztanowa 3, 41-200 Sosnowiec, Poland; ^2^Department of Clinical Chemistry and Laboratory Diagnostics, Medical University of Silesia, ul. Kasztanowa 3, 41-200 Sosnowiec, Poland; ^3^Center of Experimental Medicine, Medical University of Silesia, ul. Kasztanowa 3, 41-200 Sosnowiec, Poland; ^4^Department of Statistics, Medical University of Silesia, ul. Kasztanowa 3, 41-200 Sosnowiec, Poland

## Abstract

Changes in extracellular matrix glycosaminoglycans during the wound repair allowed us to apply the burn model in which therapeutic efficacy of propolis and silver sulfadiazine was compared. Burns were inflicted on four pigs. Glycosaminoglycans isolated from healthy and burned skin were quantified using a hexuronic acid assay, electrophoretic fractionation, and densitometric analyses. Using the reverse-phase HPLC the profile of sulfated disaccharides released by chondroitinase ABC from chondroitin/dermatan sulfates was estimated. Chondroitin/dermatan sulfates and hyaluronic acid were found in all samples. Propolis stimulated significant changes in the content of particular glycosaminoglycan types during burn healing. Glycosaminoglycans alterations after silver sulfadiazine application were less expressed. Propolis maintained high contribution of 4-O-sulfated disaccharides to chondroitin/dermatan sulfates structure and low level of 6-O-sulfated ones throughout the observed period of healing. Propolis led to preservation of significant contribution of disulfated disaccharides especially 2,4-O-disulfated ones to chondroitin sulfates/dermatan sulfates structure throughout the observed period of healing. Our findings demonstrate that propolis accelerates the burned tissue repair by stimulation of the wound bed glycosaminoglycan accumulation needed for granulation, tissue growth, and wound closure. Moreover, propolis accelerates chondroitin/dermatan sulfates structure modification responsible for binding growth factors playing the crucial role in the tissue repair.

## 1. Introduction

Local burn treatment is based on the application of silver sulfadiazine (AgSD) which, since its introduction into clinical practice by Fox, has been the agent of choice for topical burn therapy [1]. AgSD is an effective agent in controlling the infection of the burned skin. Unfortunately, evidence exists that the use of AgSD places patients at increased risk of many undesirable effects. It was also found that AgSD may lead to prolongation of the wound reepithelialization process as well as to decreased mechanical strength of the dermal tissue [2]. The previous side effects were not observed after burns treatment with propolis. According to clinical and histopathological assessments, propolis accelerates regenerative and reconstructive processes [3]. Wound healing is a dynamic, interactive process involving precisely interrelated phases, that is, hemostasis, inflammation, proliferation, and tissue remodeling, overlapping in time as well as maintaining tissue integrity [4, 5]. The healing process results from the interaction between different cell types and extracellular matrix (ECM) components, such as glycosaminoglycans (GAGs) [6]. Sulfated GAGs, that is, chondroitin/dermatan sulfates (CS/DS), heparan sulfates, and heparin (HS/H) as well as keratan sulfates (KS), are covalently attached to protein core forming proteoglycans (PGs). Hyaluronic acid (HA), which is an unsulfated GAG, does not form covalent links with proteins [7, 8]. GAGs play a key role in each phase of wound healing by stimulation of cell migration, differentiation, and proliferation as well as regulation of ECM organization and metabolism [8]. The aim of the present study was to compare the therapeutic efficacy of the silver sulfadiazine and propolis in the treatment of minor skin burns inflicted on white domestic pigs by GAGs analysis. 

## 2. Material and Methods

### 2.1. Therapeutic Agents

Propolis ointment preparation (ApiMED, Poland) accepted by the National Institute of Hygiene (certificate number: HZ/06107/00, date: 11.04.2000). 1% silver sulfadiazine cream (AgSD), Lek, Poland. 

### 2.2. Tissue Material

The study protocol was approved by the Ethics Committee of the Medical University of Silesia. Four 16-week-old domestic pigs have been chosen as useful experimental animals for the evaluation of wound repair because of many similarities of pig skin to human one such as thickness and structure of epidermis and dermis, the structure of the dermoepidermal junction, subcutaneous tissue structure, or the number and distribution of blood vessels [9, 10]. In addition, human and pig skin exhibits many similarities in terms of proliferation time of epithelial cells, type of keratinous proteins synthesized by epithelial cells, and lipid composition of the stratum corneum [10]. 72 burn wounds were inflicted according to Hoekstra et al. [9] standard model. Pigs were housed in accordance with G.L.P. standards of Polish Veterinary Law. Animals were divided into two groups—a control one and an experimental one—each of them containing two animals. Control wounds were treated with physiologic saline (9 mg/mL NaCl; Polfa Lublin, Poland) to observe the healing process occurring without management (one animal) or with propolis vehicle (ApiMED Poland) (another animal) twice a day in order to exclude its possible effect on the propolis during the whole experiment. Burns were treated with propolis (one animal) or AgSD (another animal)—twice a day from the first to the twenty-first day of the study. Biopsies, in three replications, were taken from normal skin (day “0”) and from the same wound on postburn days—3rd, 5th, 10th, 15th, and 21st.

### 2.3. Extraction and Determination of Tissue GAGs

GAGs isolation was carried out according to Scott [11] and Van Amerongen et al. [12]. Briefly, 100 mg of tissue samples after homogenization with acetone (POCH, Poland) and weighting was digested with papain (Sigma-Aldrich, USA) to release GAG chains from PG core proteins. Both peptides generated by papain action and protein resistant to the enzyme were removed by precipitation with trichloroacetic acid (Ubichem Plc, MK). Subsequently, GAGs were dialyzed, precipitated with ethanol (POCH, Poland), dissolved in potassium acetate (POCH, Poland), and reprecipitated. The total amount of GAGs was quantified by a hexuronic acid assay [13].

### 2.4. Assay of Tissue GAGs

Samples of isolated GAGs were submitted to electrophoresis on cellulose acetate (Serva Germany), before and after the use of enzymes specifically eliminating particular GAG types, that is, chondroitinase ABC (pH 6.0) and chondroitinase ABC (pH 8.0) (obtained from Sigma-Aldrich, USA) [14]. Electrophoretic fractionation of GAGs was performed as described by Komosinska-Vassev et al. [15]. 

### 2.5. Analysis of CS/DS Sulfation Patterns

Before the analysis of sulfation patterns CS/DS isolated from variously treated wounds were depolymerized by chondroitinase ABC in 0.05 M Tris-HCl (Sigma-Aldrich, USA) buffer, pH 8.0, for 24 h at 37°C. Obtained disaccharides were further tagged with fluorophore 2-aminoacridone (AMAC) (Sigma-Aldrich, USA) according to the method of Deakin and Lyon [16]. Briefly, disaccharides were dissolved in 10 *μ*L of 0.1 M AMAC solution in 85% DMSO/15% acetic acid (Sigma-Aldrich, USA). After 20 minutes 10 *μ*L of 1 M sodium cyanoborohydride (Sigma-Aldrich, USA) was added to the disaccharide samples which were incubated in the dark for 18 hours at room temperature. Then, fluorophore labeled disaccharides were diluted with mixture of water and 85% DMSO/15% acetic acid (1 : 1) and subjected to reverse-phase high performance liquid chromatography (RP HPLC) according to Deakin and Lyon [16] on PLRP-S 300 Å column (4.6 mm × 150 mm; Polymer Laboratories, Varian, Shropshire, UK) equilibrated in solution A (0.1 M ammonium acetate, POCH, Poland), running on a Varian ProStar HPLC system. After 2 mL gradient of 0%–10% solution B (100% methanol POCH, Poland), the disaccharides were eluted over 50 mL linear gradient of 10%–30% solution B at a flow rate of 1 mL/min. Then, short and steep 3 mL gradient of 30%–100% solution B was used. Disaccharides were detected by inline fluorescence (excitation at 425 nm and emission at 520 nm). However, due to various labeling efficiency of differently sulfated disaccharides, fluorescent disaccharide peak areas differ from real content of particular disaccharides within a mixture [16]. Thus, the obtained results were corrected by a multiplication of respective peak areas with appropriate experimental factors [16].

### 2.6. Statistical Analysis

Statistical differences between groups were determined by a multivariate analysis of variance (ANOVA), followed by Tukey's post hoc tests, accepting *P* < 0.05 as significant.

## 3. Results

**Figure 1:**
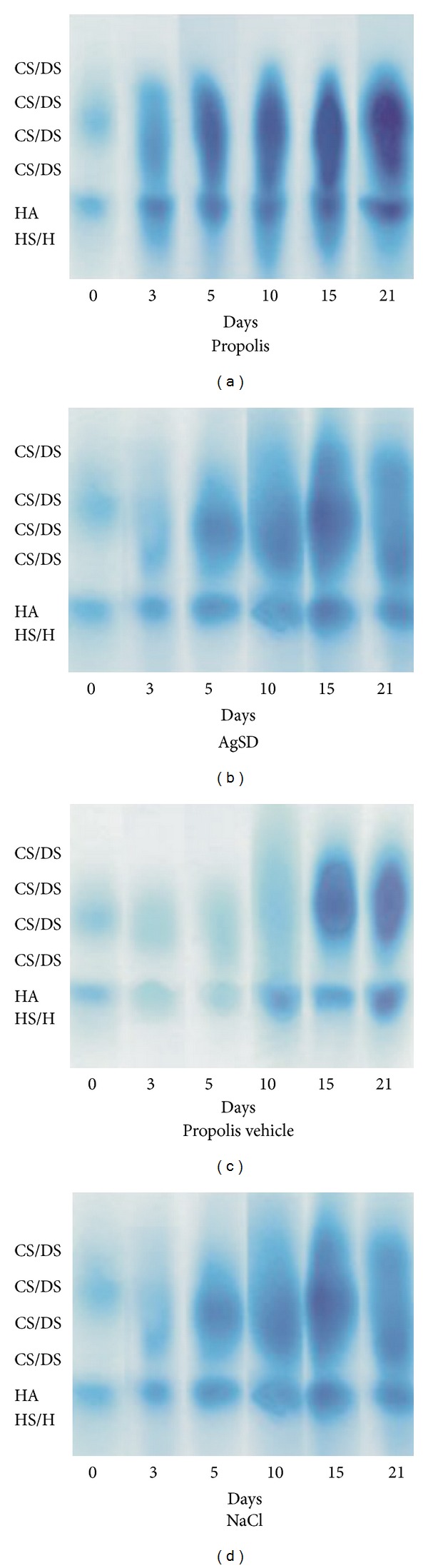
Electrophoresis of intact glycosaminoglycans (CS/DS, HA, and HS/H) isolated from normal skin (day 0) and skin samples taken from the healing wounds (postburn days 3rd, 5th, 10th, 15th, and 21st) treated with propolis (a), AgSD (b), propolis vehicle (c), and NaCl (d).

The electrophoretic analyses of tissue GAGs (Figure 1) allowed to identify CS/DS, HA, and HS/H. 

**Figure 2:**
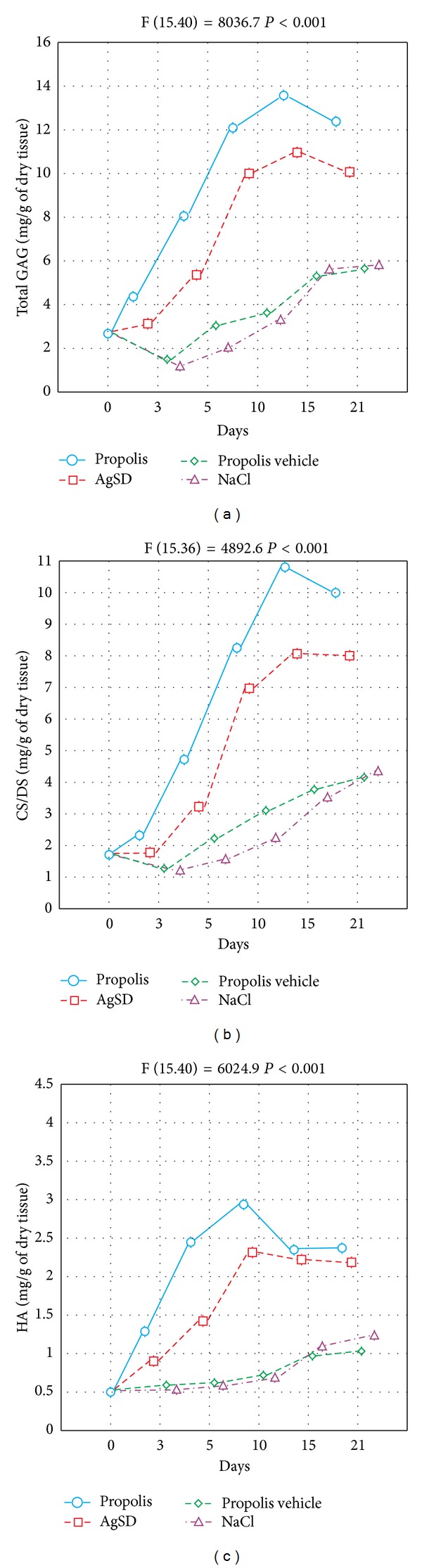
The dynamics of total GAG (a), CS/DS (b), and HA (c) content changes in skin samples taken from the healing wounds (postburn days 3rd, 5th, 10th, 15th, and 21st) treated with propolis, AgSD, propolis vehicle, and NaCl. Day 0—normal skin. The data were analyzed by ANOVA.

The expression of HS/H constituting a small amount of wound matrix total GAGs [6] was precisely discussed in our previous work [17]. As can be seen from Figure 2(a) the total amount of GAGs increased in the wound bed treated with propolis, AgSD, NaCl, and propolis vehicle. However, the most marked alteration was found in regard to apitherapeutic agent application. In the final phase of the experiment (days 15–21), the total GAGs content decreased after propolis and AgSD implementation. All the alterations were statistically significant. The majority of GAGs were identified as CS/DS. An increase in the CS/DS content during the healing process (days 0–15th), particularly visible after propolis treatment, was followed by the reduction in this fraction amount on the 15th day of the study.

**Figure 3:**
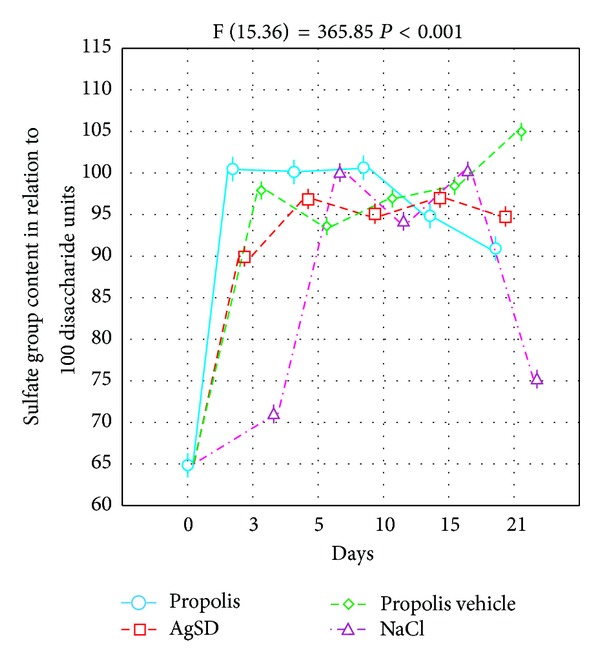
Dynamics of the CS/DS sulfation degree alterations in skin samples taken from the healing wounds (postburn days 3rd, 5th, 10th, 15th, and 21st) treated with propolis, AgSD, propolis vehicle, and NaCl. Day 0—normal skin. Sulfation degree was calculated as the sulfate group content in relation to 100 disaccharide units on the basis of disaccharide profiles generated by chondroitinase ABC action on CS/DS as described in Section 2. The data were analyzed by ANOVA.

**Figure 4:**
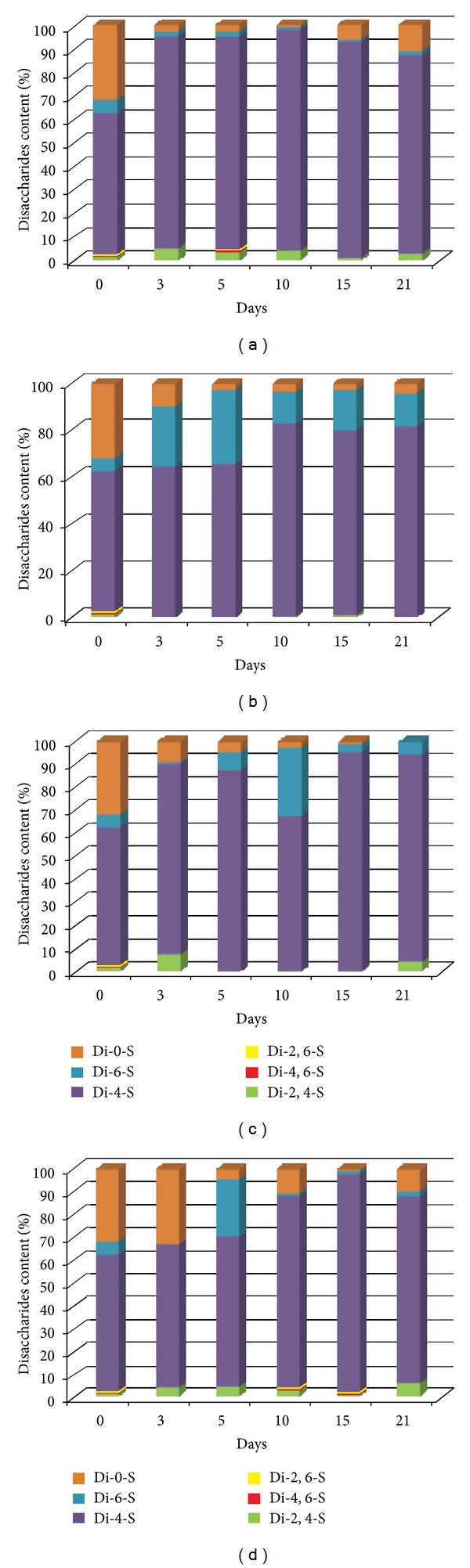
Dynamics of the sulfate disaccharide profile alterations in CS/DS chains from healing wounds (postburn days 3rd, 5th, 10th, 15th, and 21st) treated with propolis (a), AgSD (b), propolis vehicle (c), and NaCl (d). Day 0—normal skin. Disaccharides were released from CS/DS by chondroitinase ABC and subjected to reverse-phase HPLC after labeling with fluorophore 2-aminoacridone as described in Section 2.

When AgSD was applied, the CS/DS content was growing until the 15th of the experiment. However, it did not change at the end of the study. NaCl as well as propolis vehicle led to moderate elevation of the CS/DS content. The differences in the CS/DS content between the first and the last day of the experiment were statistically significant. The results obtained are presented in Figure 2(b).  Figure 2(c) shows that the burn healing process results in significant changes of wound bed HA. The most marked increase in HA content followed by the reduction (both alterations statistically significant) and subsequent stability was observed in the site of injury after propolis treatment. The similar changes were found when AgSD was applied. Statistically significant growing tendency in HA changes was displayed by healing tissues treated with NaCl and propolis vehicle. Our examination revealed that the CS/DS are the main GAGs of healing postburn lesions irrespective of used agents. However, question arises whether used medication of wounds may affect the CS/DS sulfation pattern which determines the binding potential of these molecules and so can modulate the repair process. Thus, to address this issue we have examined by reverse-phase HPLC the profile of sulfated disaccharides released by chondroitinase ABC from CS/DS derived from variously treated postburn wounds. The obtained results are presented in Figures 3 and 4. 

As can be seen from Figures 3 and 4, the sulfation pattern characterizing CS/DS synthesized in the course of physiological repair reflected in lesions treated with NaCl shows marked remodeling when compared to sulfation pattern of normal skin CS/DS. This is reflected in the remarkable increase in these GAG sulfation degrees starting from the 5th day of healing and persisting at least until 21st day. The CS/DS oversulfation results initially from transient enhancement of 6-O-sulfated disaccharide number replaced from 10th day by augmentation of 4-O-sulfated disaccharide content which attains the peak on 15th day of healing. Moreover, 2–4-fold increase in contribution of disulfated disaccharides, particularly 2,4-O-disulfated ones, to the structure of CS/DS derived from NaCl treated wounds is also observed as compared to CS/DS from normal tissue. Likewise NaCl, AgSD and propolis when used for the treatment of postburn wounds also lead to CS/DS oversulfation which appears as early as on 3rd day of healing and shows some downward trend on 21st day. However, in spite of a similar effect on total sulfation level, AgSD and propolis display distinct impact on sulfate disaccharide profile in CS/DS. The GAG oversulfation evoked by AgSD is in great part associated with a marked accumulation of 6-O-sulfated disaccharides particularly pronounced on 5th day of healing. In contrast, propolis maintains high contribution of 4-O-sulfated disaccharides to the CS/DS structure and low level of 6-O-sulfated ones throughout the observed period of healing. Interestingly, similar proportion between 4-O-sulfated and 6-O-sulfated disaccharides in the CS/DS chains derived from NaCl treated wounds is observed only on day 10th of the repair. Moreover, unlike AgSD and similarly to NaCl, propolis leads to preservation of significant contribution of disulfated disaccharides especially 2,4-O-disulfated ones to the CS/DS structure throughout the observed period of healing. All previously mentioned effects of propolis result from specific influence of propolis on CS/DS metabolism during wound healing as judged from the comparison of sulfation patterns characterizing CS/DS derived from wounds treated with propolis and propolis vehicle. 

## 4. Discussion

Changes in ECM glycosaminoglycans in the course of the healing process are generally known [6, 18]. It allowed us to apply the experimental model of tissue repair in order to compare the therapeutic efficacy of propolis with AgSD being the agent of choice for the outpatient management of minor burns [1]. The burn treatment with AgSD is known to exert side effects not observed during burn management with propolis [19]. The propolis used in the present study to treat minor skin burns is well known for its anti-inflammatory, antimicrobial, antifungal, immunomodulatory, anticancer, antioxidative, granulation tissue growth and wound closure accelerating properties [3, 20–22]. The biochemical evaluation of propolis and AgSD influence on burn healing, based on GAGs analyses, has been undertaken in the present study. We have investigated total GAGs, HA, and CS/DS accumulation and distribution during the consecutive phases of wound healing. It has been found that the total GAGs content in burn wounds treated with propolis is growing up to the fifteenth day eventually being slightly reduced at the end of the experiment. A similar tendency of the GAGs amount modifications was demonstrated after AgSD application. The total GAGs content alterations in the course of healing process, particularly visible following propolis application, correspond with the glycan changes during the wound repair reported by Bentley [23] and Hoffman et al. [24]. We propose that the observed changes in the total GAGs content after propolis application may be connected with the ability of its flavonoid compounds to reduce lipid peroxidation and prevent necrosis of cells such as fibroblasts [25]. Mentioned cells are responsible for GAGs synthesis in the course of healing process [26]. We suggest that the elevated amount of total GAGs in wounds following propolis application promotes the repair process. It is known that GAGs play an active role during wound healing [8] by regulating cellular adhesion, migration, and proliferation [7]. Mentioned functions are connected with GAGs and PGs ability to bind and modulate a vast repertoire of proteins, that is, growth factors, cytokines, morphogens, and enzymes [27]. The most common skin GAG is DS [28] being simultaneously the major glycan in wound fluid [29]. During wound healing DS activates endothelial leukocyte adhesion by stimulation of ICAM-1 [30] or promotion fibroblast growth factor-2, which is also involved in the interaction with hepatocyte growth factor/scatter factor [31], heparin cofactor II, platelet factor 4, fibronectin, and protein C inhibitor [32]. In addition to DS, the other glycosaminoglycan—CS [32]—occurs in normal skin in smaller amounts [33]. Upregulated CS expression [34] during wound repair seems to be connected with the mentioned glycan ability to mediate FGF-2-induced cell proliferation, regulate cell adhesion, and enhance cell spreading and migration by activating focal adhesion of growth factor [8]. However, DS often occurs in copolymeric form with CS [35]; therefore, we decided to estimate the expression of mentioned GAGs in wound matrix together with CS/DS. It has been found that propolis stimulates CS/DS accumulation in wounds in a greater degree than AgSD does. The results obtained are in agreement with those described by Siméon et al. [6]. They observed that wounds treatment with complex Gly-his-lys-Cu^2+^ (GHK-Cu) led to the elevation in mentioned glycan amount. We suggest that propolis action may resemble that of GHK-Cu described as a growth factor for differentiated cells, a chemotactic agent for monocytes/macrophages and mast cells supporting angiogenesis and enhancing the expression of ECM macromolecules [6]. In the course of tissue repair not only is the amount of CS/DS, expressed in the wound bed, important but also GAGs sulfation pattern [36]. It is generally accepted that the sulfation pattern of GAGs determines their binding potential [35, 37], though detailed requirements as to GAG structure implicated in particular activities are yet poorly known. On the other hand, our study is the first one which examines the effect of propolis on sulfation pattern of CS/DS. We have observed that propolis stimulates the accumulation of 4-O-sulfated and 2,4-O-sulfated disaccharides in the CS/DS chains during initial phase of experimental wound repair. It is known that these disaccharides promote CS/DS binding to FGF-2, FGF-7, and/or PDGF [36, 38]. All these molecules play a crucial role in the healing process as regulators of proliferation, migration, survival, and/or secretory activity of such cells as fibroblasts, endothelial cells, and keratinocytes [38, 39]. Growth factor binding to CS/DS protects the regulators from proteolysis and/or concentrates them near their cell receptors [36, 37]. In addition, CS/DS abundant in 4-O-sulfated and/or 2,4-O-disulfated disaccharides are also able to stimulate FGF-7 and FGF-2 mediated cell proliferation functioning as the growth factor coreceptors [38]. Thus, it seems that the application of propolis to wound treatment, via the influence on CS/DS sulfation, can accelerate wound reepithelialization and granulation. An abundant component of wound environment is HA [18]—a structural molecule which provides tissue hydration, acts as a signaling molecule, interacts with cell surface receptors, and promotes cell proliferation, migration, differentiation, and gene expression [40]. The most marked increase in HA content, observed in the case of propolis treatment, was followed by the reduction in HA amount at the final stage of the experiment. Propolis stimulation of the HA content may be connected with the ability of the apitherapeutic agent to enhance the expression of TGF-*β* [41] which, in turn, stimulates fibroblasts to synthesize HA [42]. Our results also seem to be in agreement with those of Siméon et al. [6] suggesting that propolis, similarly as GHK-Cu, induces the glycan transformation. The propolis influence on ECM may be connected with the ability of its compounds such as galangin and caffeic acid phenethyl ester (CAPE) to prevent the inhibition of GAG synthesis during inflammation. CAPE also stimulates wound reepithelization and increases keratinocyte proliferation and thickness of the wound epidermis [43]. Our previous studies showed that propolis accelerates regenerative and reconstructive processes, reduces wound healing time, and exerts a beneficial influence on animal general condition. We observed the wound cleaning process accompanied by the ground substance production, blood vessel growth leading to granulation tissue formation as well as collagen fibers maturation contributing to the scar formation [3]. Moreover, we also previously found that propolis applied in burn wound treatment displayed higher antimicrobial efficacy than AgSD [44]. The results of different examinations have demonstrated that propolis is an active, alternative therapeutic agent to treat skin injuries presenting antimicrobial activity and favorable influence in the course of wound healing process [25].

## 5. Conclusion 

Our findings demonstrate that propolis accelerates the burned tissue repair by stimulation of the wound bed GAGs (CS/DS and HA) accumulation needed for granulation, tissue growth and wound closure. Moreover, we have found that propolis accelerates CS/DS structure modification responsible for binding various growth factors—regulatory molecules playing a crucial role in the tissue repair.
